# The Histidine Decarboxylase Gene Cluster of *Lactobacillus parabuchneri* Was Gained by Horizontal Gene Transfer and Is Mobile within the Species

**DOI:** 10.3389/fmicb.2017.00218

**Published:** 2017-02-17

**Authors:** Daniel Wüthrich, Hélène Berthoud, Daniel Wechsler, Elisabeth Eugster, Stefan Irmler, Rémy Bruggmann

**Affiliations:** ^1^Interfaculty Bioinformatics Unit and Swiss Institute of Bioinformatics, University of BernBern, Switzerland; ^2^Agroscope, Institute for Food SciencesBern, Switzerland

**Keywords:** histamine, *Lactobacillus parabuchneri*, histidine decarboxylase, genomic island, mobile elements, HDC gene cluster

## Abstract

Histamine in food can cause intolerance reactions in consumers. *Lactobacillus parabuchneri* (*L. parabuchneri*) is one of the major causes of elevated histamine levels in cheese. Despite its significant economic impact and negative influence on human health, no genomic study has been published so far. We sequenced and analyzed 18 *L. parabuchneri* strains of which 12 were histamine positive and 6 were histamine negative. We determined the complete genome of the histamine positive strain FAM21731 with PacBio as well as Illumina and the genomes of the remaining 17 strains using the Illumina technology. We developed the synteny aware ortholog finding algorithm SynOrf to compare the genomes and we show that the histidine decarboxylase (HDC) gene cluster is located in a genomic island. It is very likely that the HDC gene cluster was transferred from other lactobacilli, as it is highly conserved within several lactobacilli species. Furthermore, we have evidence that the HDC gene cluster was transferred within the *L. parabuchneri* species.

## Introduction

Biogenic amines are basic nitrogenous compounds found in foods and in beverages. They are synthesized by the decarboxylation of amino acids. Among the various biogenic amines, histamine is of particular interest, as ingestion can cause intolerance symptoms such as gastrointestinal disorders, rhinorrhea, headache, and pruritus (Maintz and Novak, 2007). The decarboxylation of histidine to histamine is catalyzed by histidine decarboxylase (HDC, EC 4.1.1.22). In bacteria, two families of HDC have been identified and characterized. One family requires pyridoxal 5′-phosphate as co-factor and is found in Gram-negative bacteria which often play a role in the formation of histamine in fish. The other family possesses instead of pyridoxal 5′-phosphate a covalently bound pyruvoyl moiety as prostetic group and is present in Gram-positive bacteria (Landete et al., 2008). The formation of histamine in fermented food such as cheese and wine is related to Gram-positive bacteria. Very well documented are the HDCs from *Oenococcus oeni* (formerly known as *Leuconostoc oenos*) and *Lactobacillus hilgardii* isolated from wine (Coton et al., 1998; Lucas et al., 2005). In bacterial isolates from cheese, HDC enzymes were identified *in Lactobacillus buchneri* and *Streptococcus thermophilus* (Martín et al., 2005; Rossi et al., 2011). When *L. buchneri* St2A and *L. buchneri* Lb14 were used as adjuncts in cheese making, the cheeses developed high levels of histamine (Sumner et al., 1985; Joosten and Northolt, 1989; Choudhury et al., 1990). Meanwhile, both strains, which are deposited in the Belgian coordinated Collection of Microorganisms and German Collection of Microorganisms and Cell Cultures, were re-identified as *Lactobacillus parabuchneri* (*L. parabuchneri* LMG 11773 and *L. parabuchneri* DSM 5987).

We repeatedly isolated strains of *L. parabuchneri* from various cheeses containing elevated histamine concentration (Berthoud et al., 2016). Most of the strains synthesized histamine when incubated with histidine. When cheese was inoculated with the histamine producing *L. parabuchneri* FAM21731 the concentration of histamine increased during cheese ripening (Fröhlich-Wyder et al., 2013). Additionally, we observed that *L. parabuchneri* produced 1,2-propanediol, acetate, ammonia and carbon dioxide (Fröhlich-Wyder et al., 2013). These metabolites influence cheese quality as they cause an increase of the pH and eye formation. Consequently, we consider this bacterium, especially the histamine-producing strains, as a potential spoilage organism in cheese production. The aim of this study was to investigate the genome variability of this species by comparing genomes of various strains isolated from cheese and milk.

## Materials and methods

### Strains, culture media, and formation of histamine

The *L. parabuchneri* strains (Table 1) used in this study were cultivated in MRS broth (de Man et al., 1960) at 30°C. For longterm storage, strains were stored at −80°C in sterile resonstituted skim milk powder (10%, w/v). To determine the formation of histamine, the strains were cultivated in MRS broth supplemented with 0.3% L-histidine for 3 days at 30°C. Afterwards, the culture supernatants were diluted 1:100 in methanol and 10 μL was applied to high-performance thin-layer chromatography (HPTLC) cellulose plates. After the plates had been developed with 2-propanol, 25% ammonia (3:1, v/v), the imidazole ring of histamine and histidine was visualized by dipping the plates into Pauly's reagent.

**Table 1:** **Origin and genome assembly information of the *L. parabuchneri* strains**.

**Strain**	**Origin**	**Largest scaffold [bp]**	**n50 [bp]**	**Genome size [bp]**	**No. of scaffolds**	**Accession number**	**Sequencing technology**	**Histamine formation**
FAM21731	Emmental	2,600,578	2,600,578	2,726,576	3	CP018796, , CP018798	PacBio/Illumina (101 × 101)	Positive
FAM21809	Tête de Moine	354,135	124,546	2,570,634	38	MSAS00000000	Ion Torrent/ Illumina (151 × 151)	Positive
FAM21823	Mont soleil	313,120	114,067	2,672,131	63	MSAT00000000	Illumina (151 × 151)	Positive
FAM21829	Emmental	286,113	194,211	2,738,317	66	MSAU00000000	Illumina (151 × 151)	Positive
FAM21834	Tilsit	200,713	65,547	2,762,714	108	MSAV00000000	Illumina (151 × 151)	Positive
FAM21835	Tilsit	504,044	143,467	2,665,006	61	MSBE00000000	Illumina (151 × 151)	Negative
FAM21838	Swiss Alpine cheese	307,911	53,605	2,543,799	143	MSAW00000000	Illumina (151 × 151)	Negative
FAM23163	Tête de Moine	690,536	161,307	2,567,644	38	MSAX00000000	Illumina (151 × 151)	Positive
FAM23164	Tête de Moine	363,950	132,527	2,704,805	55	MSAY00000000	Ion Torrent/ Illumina (151 × 151)	Positive
FAM23165	Tête de Moine	363,950	132,527	2,706,330	60	MSAZ00000000	Ion Torrent/ Illumina (151 × 151)	Positive
FAM23166	Tête de Moine	363,642	115,983	2,708,155	60	MSBA00000000	Ion Torrent/ Illumina (151 × 151)	Positive
FAM23167	Tête de Moine	363,642	70,556	2,654,853	164	MSBB00000000	Ion Torrent/ Illumina (151 × 151)	Positive
FAM23168	Tête de Moine	690,536	161,307	2,566,645	36	MSBC00000000	Illumina (151 × 151)	Positive
FAM23169	Tête de Moine	351,075	76,021	2,799,109	130	MSBD00000000	Illumina (151 × 151)	Positive
FAM23279	Raw milk	337,373	192,222	2,611,434	84	MSBF00000000	Illumina (151 × 151)	Negative
FAM23280	Raw milk	330,747	192,349	2,563,148	65	MSBG00000000	Illumina (151 × 151)	Negative
FAM23281	Raw milk	330,747	192,350	2,616,211	79	MSBH00000000	Illumina (151 × 151)	Negative
FAM23282	Raw milk	437,069	188,636	2,613,937	54	MSBI00000000	Illumina (151 × 151)	Negative

### Library preparation and sequencing

DNA was extracted from *L. parabuchneri* with ZR Fungal/Bacterial DNA MiniPrep Kit (Lucerna Chem AG, Lucerne; Switzerland) and concentrated with Genomic DNA Clean & Concentrator Kit (Lucerna Chem AG) according to the manufacturer's instructions.

For the PacBio sequencing, 5 μg of high molecular weight DNA from *L. parabuchneri* FAM21731 were sheared in a Covaris g-TUBE (Covaris, Woburn, MA, USA) to obtain 20 kb fragments. After shearing, the DNA size distribution was analyzed with a FragmentAnalyzer (Advanced Analytical Technologies, Ames, IA, USA). A SMRTbell library was prepared using the PacBio DNA Template Prep Kit 2.0 (Pacific Biosciences, Menlo Park, CA, USA) according to the manufacturer's recommendations. The library was sequenced using two SMRT cells with P4/C2 chemistry on a PacBio RSII system with a movie length of 120 min. The sequencing yielded 118,000 post filter reads corresponding to 629 Mb with a mean read length of 5,326 bases.

FAM21731 was also sequenced using Illumina technology. The library preparation of the DNA from *Lactobacillus parabuchneri* FAM21731 using “TruSeq DNA Sample Preparation Kit” (15025064) was followed by gel size selection for 400–500 bp fragments. The library was paired-end sequenced (2 × 101 bp) in a fraction of a lane on an Illumina HiSeq 2000 instrument.

Library preparation of the DNA from the remaining 17 *L. parabuchneri* strains was performed using “TruSeq DNA PCR-Free LT Library Prep” (FC-121-3003, Insert size option: 350 bp). The libraries were paired-end sequenced (2 × 151 bp) in a fraction of a lane on an Illumina HiSeq 3000 instrument.

The strains FAM21809, FAM23164, FAM23165, FAM23166, and FAM23167 were additionally sequenced using an Ion Torrent device. Library preparation, amplification and sequencing was performed using Ion Xpress Plus Fragment Library Kit for AB Library Builder System, Ion PGM Template OT2 200 Kit and Ion PGM Sequencing 200 Kit v2 (Life Technologies Europe BV, Zug, Switzerland) according to the manufacturer's instructions. Libraries were barcoded using Ion Xpress Barcode Adapters 1-16 Kit (Life Technologies Europe BV). Five Libraries were pooled and sequenced on a single Ion 318 chip (Life Technologies Europe BV).

### *De novo* assembly

The Pacbio reads were assembled using the HGAP 3 (smrtanalysis-2.2.0) (Chin et al., 2013) standard procedure. To close the cyclic DNA of the chromosome and the plasmids, the assembly was performed twice. The resulting scaffolds of both assemblies were aligned using mauve (Darling et al., 2010) and the ends of the scaffold were replaced by the contiguous sequence of the other assembly. The assembly resulted in one chromosome (2,600,578 bp) and two plasmids (58,093 and 67,905 bp). Remapping and variant calling of the PacBio reads was performed using quiver (smrtanalysis-2.2.0) in which GATK (McKenna et al., 2010) is embedded. Finally, the kineticsTools (smrtanalysis-2.2.0) was used for the detection of modified bases in the genome. To find modified motifs MotifMaker (smrtanalysis-2.2.0) was used.

The Illumina reads were quality trimmed using Trimmomatic (version 0.33, options: SLIDINGWINDOW:4:8 MINLEN:127) (Bolger et al., 2014). The trimmed reads were assembled using SPAdes (version 3.6.1, options: –careful –mismatch-correction -k 21,33,55,77,99,127). The resulting sequences were scaffolded using SSPACE (version 3.0, default options) (Boetzer et al., 2011). Scaffolds with a lower median coverage than 20% of the median read-depth of the whole genome and scaffolds shorter than 200 bp were excluded.

### Variant detection using illumina and ion torrent reads

The reads were mapped to the assembly of PacBio reads using BWA (version 0.7.10) (Li and Durbin, 2009). The variants were determined using haplotype-caller integrated in GATK (version 3.3.0) (McKenna et al., 2010). Manual curation was performed using IGV (Thorvaldsdóttir et al., 2013).

### Construction phylogenetic trees

To locate the position of *Lactobacillus parabucheri* the protein sequence of all the proteins that are present as single ortholog in all strains were used for the construction of the phylogenetic tree. The 18 *L. parabuchneri* (Table 1) strains and also the strains downloaded from Genbank (Table S1) were annotated with Prokka (version 1.11) (Seemann, 2014) to avoid differences derived from different *ab initio* annotation tools. The orthologs were determined using OrthoMCL (version 2.0.9, default parameters) (Li et al., 2003). The amino acid sequences of the OGCs were aligned separately using clustal-omega (version 1.2.1) (Sievers et al., 2011). The tree was calculated using RAxML [version 8.1.2, (-m PROTGAMMAWAG), Stamatakis, 2006] based on the concatenated alignments.

The phylogenetic tree of the *L. parabuchneri* and *L. buchneri* strains was constructed with the same pipeline, with the difference that the nucleotide sequences of the OGCs were used and the model of RAxML was adjusted (-m GAMMAWAG).

The phylogenetic trees were visualized using iTol (Letunic and Bork, 2007).

### Pan/core genome estimation

To estimate the trend of the pan and core genome size the strains were randomly subsampled without replacement, from one to the total number of strains. For each of the 1,000 subsampling, the core and the pan genome was calculated, using an in-house python script.

### Identification and characterization of T-boxes

The T-boxes of the HDC island that were identified using Prokka (Seemann, 2014) were aligned against the T-boxes from *Lactococcus lactis* (strains SK11 and Il1403, downloaded from http://regprecise.lbl.gov) (Novichkov et al., 2013) using the MUSCLE (Edgar, 2004) web interface. The conserved regions were adapted from a previous study (Lebeer et al., 2007).

### Gene ontology (GO) term enrichment in the HDC island

GO terms were assigned to the CDSs of FAM21731 using blast2go-pipe [version 2.5, BLASTP (version 2.2.29+, NCBI non-redundant database from August 2015), Conesa et al., 2005] and InterProScan (version 5.14-53.0) (Quevillon et al., 2005).

The GO enrichment analysis was performed using the elim algorithm of the topGO package (Alexa and Rahnenführer, 2010). We took the GO terms of the genes from the HDC island as test set, and the GO terms of the remaining genes of the genome as background.

### Synteny-based ortholog finding

The SynOrF algorithm was implemented in python. The NetworkX (version 1.9.1, https://networkx.github.io/) library was used for graph analysis.

Two separate whole genome graphs were constructed based on the coding sequences (CDSs) and their positions in the genomes of the two compared strains. The CDSs were set as nodes in the graphs, and the edges were set between each CDS and its neighboring CDSs from the first to third degree in relation to genomic location. To determine homologs, all CDSs of one strain were aligned with all CDSs of the compared strain using BLASTP (version 2.2.28+) (Altschul et al., 1990). Two CDSs with an alignment similarity of more than 50% and an alignment that covered at least 50% of the longer CDSs were considered as homologs.

The selected homologs were used as edges to connect two whole genome graphs of the compared strains. Nodes of the whole genome graphs were removed if they had no edges to the other genome graph (i.e., if the CDS had no homologs in the compared genome). This removal resulted in a genome graph that consisted of unconnected subgraphs.

With the chosen homology cutoffs, it is possible for a CDS to have several homologs in the compared genome. To select the best synteny ortholog, the neighboring CDSs of the homologs in the genome graph are also taken into account (i.e., checked whether the CDS of the two genomes are also homologs). The homolog with the highest number of homolog neighbors (at least two are required) is selected as the best synteny ortholog. The whole workflow is depicted in Figure S1.

The script is available for download at https://github.com/danielwuethrich87/Synteny_ortholog_finder.git.

### Genomic island finding

Between the genome of FAM21731 and the other 17 *L. parabucheri* strains and the *L. buchneri* stains (CD034, NRRL B-30929) the synteny orthologs were identified using the SynOrF algorithm. Not conserved regions became visible after visualization of the conserved synteny othologys in a Gantt plot using ggplot2 (Wickham, 2010).

### Graphical representation

**Figure 1:**
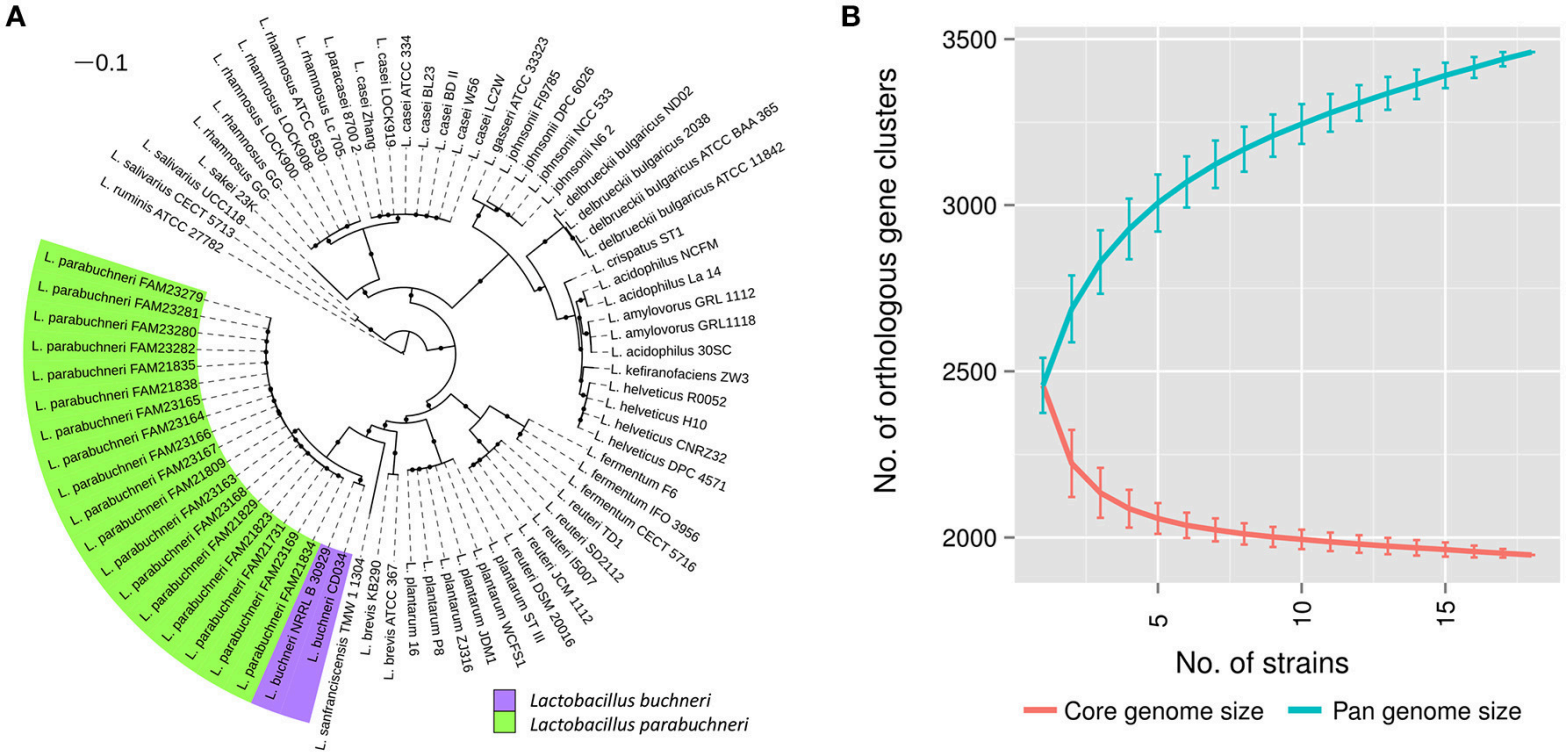
**Phylogenetic location and pan genome of *L*. *parabuchneri*. (A)** The core genome based tree shows the phylogenetic location of *L. parabuchneri* compared to other lactobacilli. The length of the lines depicts the phylogenetic distance between the strains. The black dots indicate if a branching was found in 80% or more of the bootstraps. *L. parabuchneri* strains are marked with a green and *L. buchneri* are marked with a purple background. **(B)** The solid lines represent the connections of the mean values of the core (red) and the pan (blue) genome of randomize subsampling of the 18 *L. parabuchneri* strains. The error bars indicate the standard deviation.

**Figure 2:**
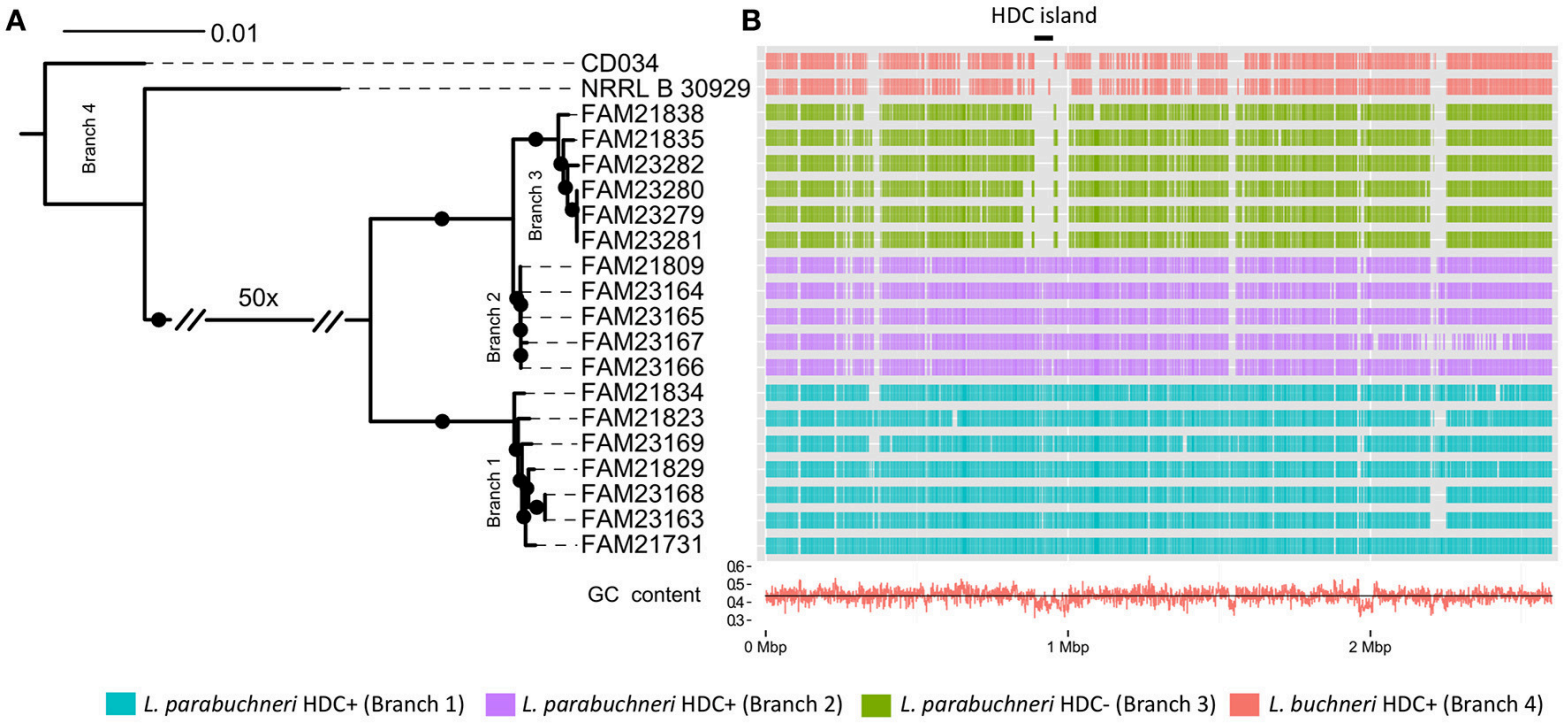
**The genomic background of histidine decarboxylase. (A)** The core genome based phylogenetic tree indicates the phylogenetic relationship of the *L. buchneri* and *L. parabuchneri* strains. The branch length indicates the phylogenetic distance and the black dots indicated branching events that are found in at least 80% of the bootstraps. **(B)** The Gantt diagram represents the synteny orthologs from the CDSs of FAM21731 that are present in other strains. The x-axis indicates the genomic location and the y-axis the strains. The black line with the label “HDC island” marks the HDC coding genomic island. The red line in the bottom indicates the GC content in the genomic regions.

**Figure 3:**
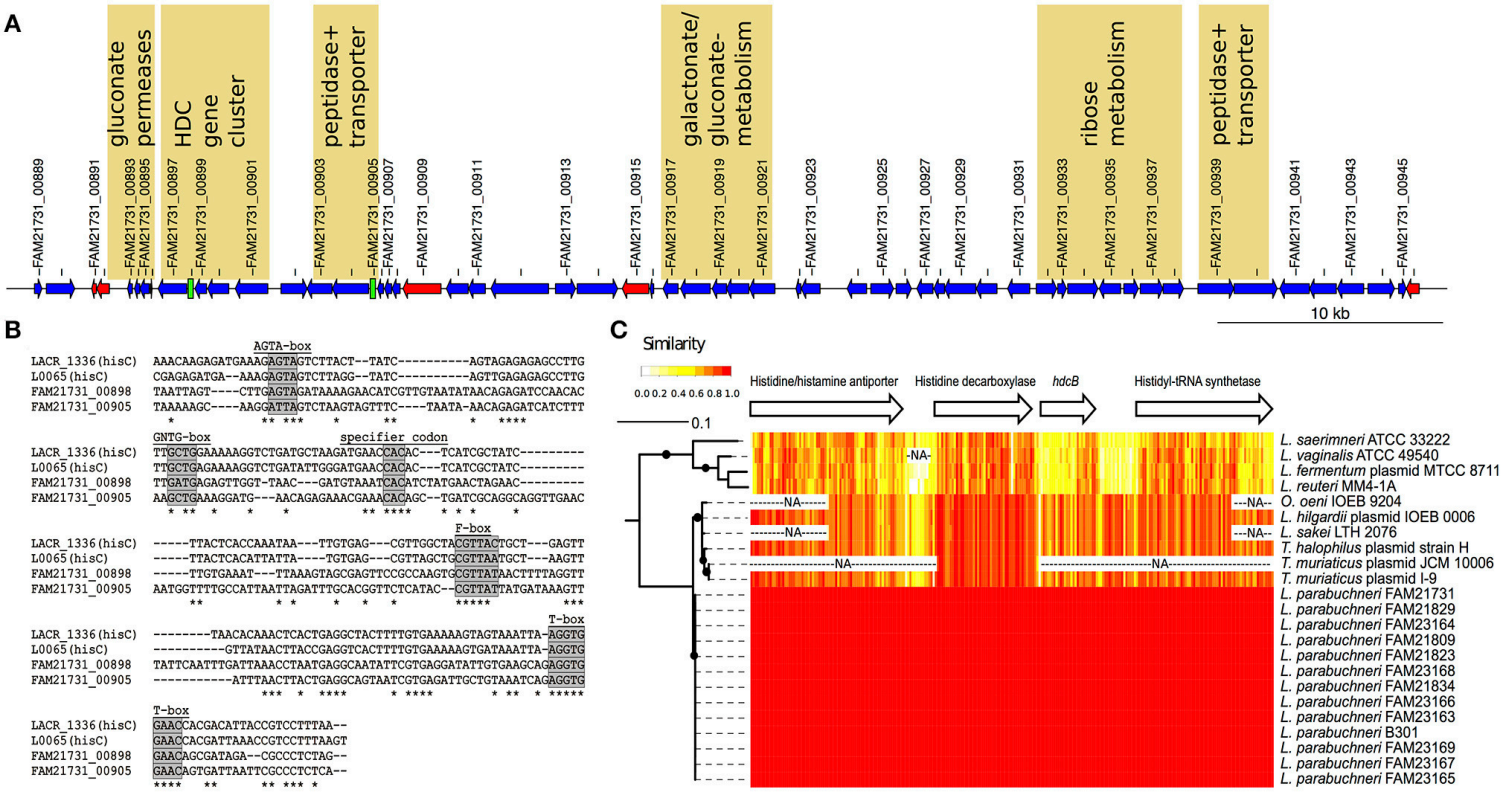
**The features of the HDC island. (A)** Schematic representation of the HDC island. The arrows indicate protein-coding sequences (blue), transposases (red), and tRNA encoding genes (black) coded on the HDC island. T-box leader sequences are depicted as green boxes. Every second genomic feature is labeled with the ID. Loci that might be related to energy production, pH-regulation and/or histidine metabolism are labeled with a yellow background. **(B)** Alignment of the histidine specific T-boxes. The T-boxes found on the HDC island were aligned to the histidine specific T-boxes from *Lactococcus lactis*. Known conserved regions in T-boxes are labeled with a gray background. **(C)** Sequence comparison of the HDC gene cluster. The phylogenetic tree is based on the amino acid sequence of the histidine decarboxylase. The branch length indicated the phylogenetic distance and the black dots branching event that were in more than 80% of the bootstraps detected. The arrows represent the length and order of the genes of the HDC gene cluster. The heat map indicates the identity (20 bp sliding window) of the DNA sequences of the different strain that carry the HDC gene cluster. The “NA” indicates sequences that are not sequenced or present in a specific strain.

**Figure 4:**
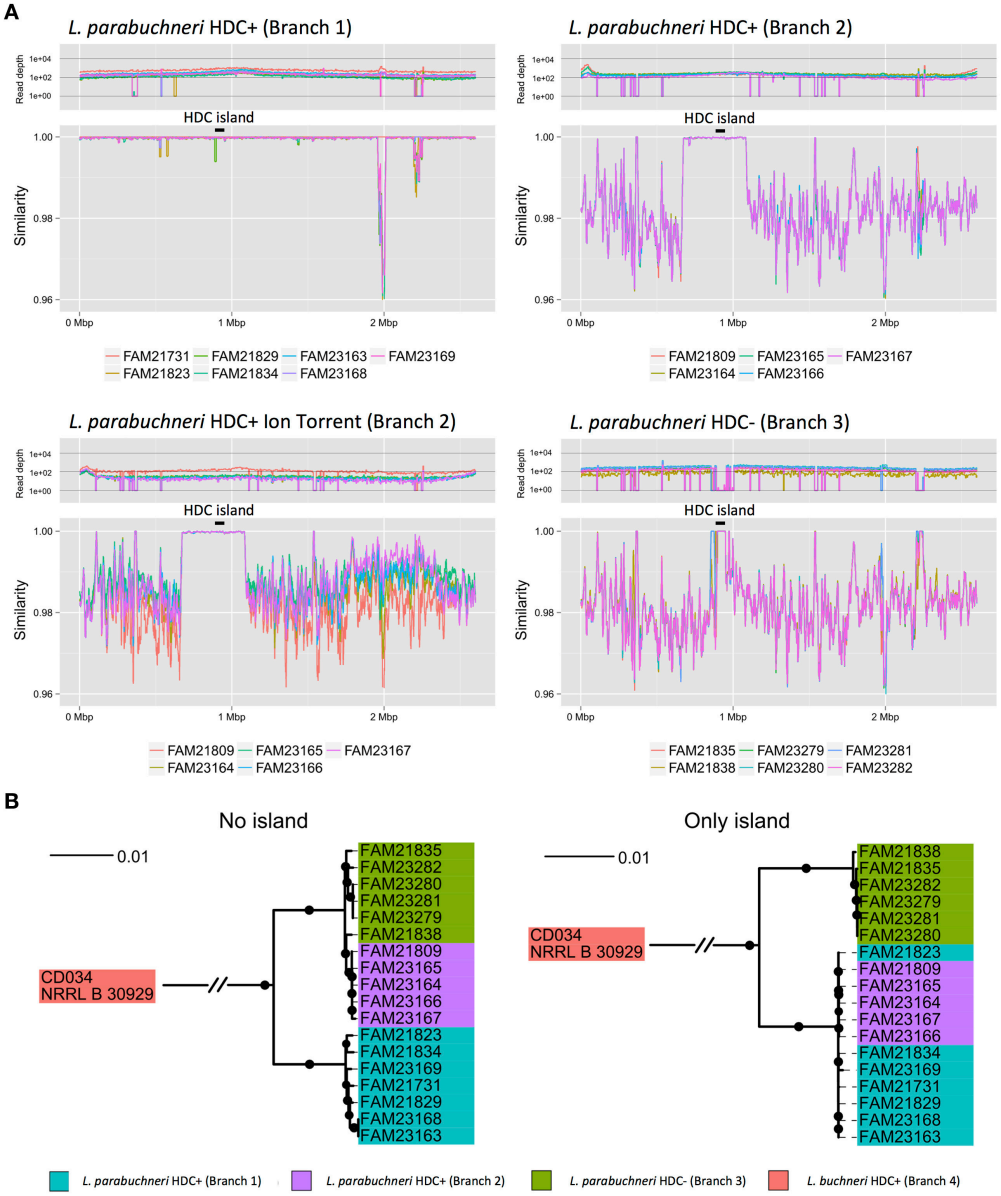
**Mobile element within *L*. *parabuchneri*. (A)** The four line plots represent the read-depth and the similarity of reads of the alignment against the FAM21731 strain. The colors indicate the origin of the reads of a strain. The three different branches of *L. parabuchneri* found in the phylogenetic analysis are depicted separately. Contrary to the strains from branch 1 and branch 3, the strains from branch 2 were not only analyzed based on Illumina reads but also Ion Torrent reads. In the strains of branch 2, a region very similar to FAM21731 is present. The black lines with the label “HDC island” mark the HDC coding genomic island. **(B)** Two phylogenetic trees were constructed based on the core genome of *L. parabuchneri* and *L. buchneri*. In the first one (no island) only the nucleotide sequences of genes that are not located on the non-conserved region were used for the tree. For the second tree (only island) only the nucleotide sequences of genes that are located on the conserved region were used for the tree construction. The branch length represents the phylogenic distance and the black dots indicate branching events that were found in at least 80% of the bootstraps.

The phylogenetic trees in Figures 1A, 2A, 3C, 4B were visualized using iTol (Letunic and Bork, 2007). The plots in Figures 1B, 2B, 3C, 4A were created with ggplot2 (Wickham, 2010). Figure S1 was drawn using Microsoft office PowerPoint 2013. Figure 3B, Figure S3 are copied text files. Figure 3A was created using genoPlotR (Guy et al., 2010).

## Results

### Genomic characterization of the *L. parabuchneri* species

Histamine in cheese is mainly formed by bacterial decarboxylation of histidine. When we analyzed cheese with elevated histamine levels (>25 mg per kg cheese), we repeatedly isolated histaminogenic strains of *L. parabuchneri* (Berthoud et al., 2016). Because a complete genome of this species was not available at the time we started the study, we selected the single molecule long read technology of PacBio to sequence the *L. parabuchneri* strain FAM21731 and to assemble a complete genome. The *de novo* assembly of the PacBio reads resulted in three contigs that we could close to circular molecules using manual correction. The largest contig is the chromosome (2,600,578 bp) and the two short contigs plasmids (58,093 bp; 67,905 bp, Table 1). Additionally, we sequenced FAM21731 using the short read technology of Illumina for quality control. Re-mapping of the PacBio and Illumina reads showed that the assembly does not have indels or base mismatches. We annotated 2,566 protein coding sequences (CDS) in the genome using Prokka (Seemann, 2014).

Besides the genome assembly, we also used the PacBio reads to identify methylated nucleotides in the genome of the strain FAM21731. We detected that adenine is methylated in the four motifs ACANNNNNNNTTYG, CACCAG, GATC and CGCCAT in 99.9, 99.9, 99.6, and 28.8% of the motifs in the genome, respectively. While the motif GATC was described in *E. coli* (Barras, 1989) and other species, so far no report about the three other motifs is available. Furthermore, all adenine in the motive ACANNNNNNNTTYG and its reverse complement CRAANNNNNNNTGT (97.0%) are methylated. The distribution of methylated motifs within the genome did not show any accumulation at specific locations.

Additionally, to the strain FAM21731, we sequenced 17 *L. parabuchneri* strains isolated from cheese and milk samples (Table 1) using Illumina technology. The assembly sizes varied between 2,562,122 and 2,799,109 bp. We annotated the genomes with Prokka and clustered the genes using OrthoMCL. Based on these gene cluster, we constructed a phylogenetic tree including—in addition to our *L. parabuchneri* strains - all complete *Lactobacillus* genomes available (57 strains, Table S1) in the Refseq database based on the OrthoMCL clusters. In total, we found 388 orthologous gene clusters (OGCs) that are present in all *L. parabuchneri* and in other *Lactobacillus* strains as single copy genes. The tree shows that the *L. parabuchneri* strains sequenced in this study cluster together with the *L. buchneri* strains (Figure 1A).

To get an insight into the gene repertoire present in *L. parabuchneri*, we constructed the pan and the core genome of the 18 sequenced strains. The core genome has a size of 1,947 OGCs and the pan genome a size of 3,461 OGCs. We applied random subsampling to the strain order to show the trend curves of the pan and core genome (Figure 1B). The size of the core genome seems to reach a plateau at 18 strains whereas the pan genome does not reach a plateau at 18 strains, i.e., additional strains would significantly increase the number of OGCs.

The phylogenetic tree shows that *L. buchneri* and its subspecies *L. parabuchneri* are very closely related (Figure 1A). We determined the size of the pan genome of the 18 *L. parabuchneri* to have 3,461 OGCs and with additional two *L. buchneri* (CD034 and NRRL B-30929) strains to have 3,910 OGCs. The small difference of 396 OGCs between the pan genomes shows that the gene content is very similar in these two species.

### The genomic background of histamine synthesis

We screened the 18 strains for their ability to synthesize histamine. Therefore, the culture supernatants of the strains were analyzed for the presence of histamine after 3 d incubation using thin-layer chromatography. We found that 12 strains produced histamine (Table 1) and that the band intensities of histamine were very similar. In these samples histidine could not be detected anymore (an example is shown in Figure S2). The other six strains, namely FAM21835, FAM21838, FAM23279, FAM23280, FAM23281, and FAM23282, did not form histamine whereas histidine was still present. This indicated that the histamine-forming strains converted histidine completely to histamine.

To link the phenotype of histamine synthesis with a genotype, we assessed the phylogenetic relationship between the histamine producing and the non-producing *L. parabuchneri* strains. These 18 strains and the two *L. buchneri* strains share 1,685 OGCs that are present in every strain as single copy genes. Based on this, we constructed a phylogenetic tree using the nucleotide sequence of these OGCs and found that the *L. parabuchneri* strains are separated into three branches (Figure 2A). We show that the phylogenetic relation and the capability to synthesize histamine correlate perfectly. Furthermore, the 12 histamine positive (HDC+) strains were separated into two branches (branch 1, branch 2) in the phylogenetic tree (Figure 2A) whereas the six histamine negative (HDC−) strains were all located on the same branch (branch 3).

The HDC gene cluster found in the chromosome of FAM21731 encodes the following proteins: A putative histidine/histamine antiporter (FAM21731_00901), a histidine decarboxylase (FAM21731_00900), a HdcB protein (FAM21731_00899) and a histidyl-tRNA synthetase (FAM21731_00897, synonym prokka annotation: Histidine–tRNA ligase). We found orthologs of these four genes in all 12 strains with the capability to synthesize histamine. The other six HDC strains lack this gene cluster completely. Sequence alignments (Figure 3C) revealed that the HDC gene cluster nucleotide sequence from *L. parabuchneri* is identical to the HDC operon of *L. buchneri* B301 (GenBank AJ749838) that was characterized by Martín et al. (2005). This indicates that strain B301 probably belongs to the *L. parabuchneri* species. Meanwhile, the nucleotide sequences of pheS (GenBank LT547856), rpoA (GenBank LT547858), and the 16S rRNA gene (GenBank LN877766) from strain B301 were deposited confirming that this strain indeed is a *L. parabuchneri* strain. Remarkably, also the histamine-forming strains LMG11773 (= St2A) and DMS 5987 (= Lb14) that originally were deposited as *L. buchneri* were re-identified as *L. parabuchneri*.

We searched the genome of FAM21731 for regions that are not conserved within the *L. parabuchneri* and *L. buchneri* strains. For this purpose, we developed an algorithm termed “Synteny Ortholog Finder” (SynOrF) (see Figure S1 and the methods section). It determines orthologs between two genomes not only based on sequence homology but also in consideration of the syntenic relationship of the genes. This pairwise comparison to identify synteny orthologs is carried out between the genome of interest and each of the remaining genomes separately. We found one 62.4 kbp long genomic region (FAM21731_00889–FAM21731_00946, Table 2) in FAM21731 that is not present in the HDC- *L. parabuchneri* and *L. buchneri* strains. This region shows a significant difference in GC-content compared to other regions of the genome (39.5 vs. 43.7%), indicating that it is a genomic island that was gained by horizontal gene transfer. We refer to this region as HDC island (HDC island, Figure 2A).

**Table 2:** **Annotation of the loci found in the HDC island**.

**Locus tag**	**Predicted function**
FAM21731_00889	DNA methyltransferase
FAM21731_00890	Hypothetical protein
FAM21731_00891	Transposase
FAM21731_00892	Transposase
FAM21731_00893	Gluconate permease
FAM21731_00894	Gluconate permease
FAM21731_00895	Gluconate permease
FAM21731_00896	tRNA
FAM21731_00897	Histidyl-tRNA synthetase with T-box leader sequence (FAM21731_00898)
FAM21731_00899	HdcB
FAM21731_00900	Histidine decarboxylase
FAM21731_00901	Arginine/agmatine antiporter
FAM21731_00902	Major facilitator superfamily transporter
FAM21731_00903	Peptidase, M20 family
FAM21731_00904	Peptide abc transporter substrate-binding protein with T-box leader sequence (FAM21731_00905)
FAM21731_00906	Hypothetical protein
FAM21731_00907	Hypothetical protein
FAM21731_00908	Hypothetical protein
FAM21731_00909	Transposase
FAM21731_00910	2-keto-3-deoxygluconate transporter
FAM21731_00911	Short-chain dehydrogenase
FAM21731_00912	Bacterial Ig-like domain (group 3)
FAM21731_00913	Methylase
FAM21731_00914	Methylase
FAM21731_00915	Transposase
FAM21731_00916	Hypothetical protein
FAM21731_00917	Transcrition regulator
FAM21731_00918	Gluconate permease
FAM21731_00919	2-dehydro-3-deoxyphosphogluconate aldolase
FAM21731_00920	2-dehydro-3-deoxygalactonokinase
FAM21731_00921	Galactonate dehydratase
FAM21731_00922	Hypothetical protein
FAM21731_00923	Hypothetical protein
FAM21731_00924	DNA modification methylase
FAM21731_00925	DNA methyltransferase
FAM21731_00926	Hypothetical protein
FAM21731_00927	Transcriptional regulator, GntR family
FAM21731_00928	Acetyltransferase
FAM21731_00929	C4-dicarboxylate abc transporter
FAM21731_00930	Ribonucleoside hydrolase
FAM21731_00931	Oxidoreductase
FAM21731_00932	Ribokinase
FAM21731_00933	Ribose pyranase
FAM21731_00934	Sugar:proton symporter
FAM21731_00935	DNA-binding transcriptional regulator
FAM21731_00936	Deoxyribose-phosphate aldolase
FAM21731_00937	Deoxyribose mutarotase
FAM21731_00938	Ribokinase
FAM21731_00939	ABC-type oligopeptide transport system, periplasmic component
FAM21731_00940	Dipeptidyl aminopeptidase
FAM21731_00941	Acetylornithine deacetylase
FAM21731_00942	Amidohydrolase
FAM21731_00943	Amidohydrolase
FAM21731_00944	Hypothetical protein
FAM21731_00945	Cell wall anchor protein
FAM21731_00946	Transposase

We applied gene ontology (GO) enrichment with the genes coded in the HDC island and found that the GO terms gluconate transmembrane transport (GO:0035429, *p* = 1.7e-06), D-ribose metabolic process (GO:0006014, *p* = 2.8e-04) and histidine metabolic process (GO:0006547, *p* = 8.0e-04) are enriched with a high significance (*p* < 0.001). The genes related to histidine metabolic process are the four CDSs of the HDC gene cluster. The analysis of the gluconate transmembrane transport revealed that a cluster of three neighboring gluconate permeases on the HDC island is directly adjacent to the HDC gene cluster and a cluster of five genes involved in gluconate and galactonate (a stereoisomer of gluconate) metabolism (Figure 3A). The functional annotation shows that the genes involved in the ribose metabolism are arranged in a cluster of seven neighboring genes (Figure 3A). Finally, we identified two T-boxes on this genomic island that showed high similarity to two histidine-specific T-boxes of *Lactococcus lactis* (Figure 3B). One of these T-boxes (FAM21731_00898) is upstream of the histidyl-tRNA synthetase, the fourth gene of the HDC gene cluster. The second T-box (FAM21731_00905) is upstream of a gene cluster that consists of a peptidase (FAM21731_00903) and a peptide transporter (FAM21731_00904). We also found a second gene cluster consisting of a peptide transporter (FAM21731_00939) and a peptidase (FAM21731_00940) within the HDC island (Figure 3A).

We determined single nucleotide polymorphisms (SNPs) between the different strains and FAM21731. We found a 400 kbp region of the FAM21731 genome (FAM21731_00682–FAM21731_01148) that was conserved within the strains of branch 1 and branch 2 but not of branch 3 (Figure 4A). Interestingly, the HDC island is located within this 400 kbp region. Phylogenetic analysis of the *L. parabuchneri* strains only including the CDSs from 400 kbp region showed a clustering of the strains from branch 1 and branch 2 into a single branch (Figure 4B). Contrary, the phylogenetic analysis based on the CDS of the core genome, excluding the genes of the 400 kbp region, showed a clustering of the strains from branch 2 and branch 3 into a single one (Figure 4B).

### Comparison of the HDC gene clusters to other species

*L. parabuchneri* is not the only species that can synthesize histamine. The HDC gene cluster found in *L. parabuchneri* shows the same gene order as the ones in *Tetragenococcus halophilus, Tetragenococcus muriaticus, Lactobacillus hilgardii, Lactobacillus sakei, Lactobacillus saerimneri, Lactobacillus vaginalis, Lactobacillus fermentum, Oenococcus oeni* and *Lactobacillus reuteri*. Gene clusters from species with different gene order (e.g., *S. thermophilus*) were not included. We observed that the nucleotide sequence of the gene encoding the histidine decarboxylase is closely related to the ones of these species (Figure 3C) (74.7–89.2% sequence identity). Comparing these results to the phylogeny of the core genome of the lactobacilli, we observed that the histidine decarboxylases are more closely related (e.g., *L. sakei*) to one another than the core genomes (Figure 1A).

Our analyses showed that the HDC gene cluster is located in the chromosome of *L. parabuchneri* as well as *L. reuteri* (GenBank NC_009513). In *L. vaginalis*, the HDC locus is in a 693-kb scaffold (GenBank NZ_GG693412). The scaffold size indicates that the HDC is probably also chromosomal. This hypothesis is supported by the observation that no plasmids were found in histamine-producing *L. vaginalis* strains (Diaz et al., 2015). On the contrary, the HDC gene cluster was found in plasmids in strains of *O. oenii* (Lucas et al., 2005), *L. hilgardii* (Lucas et al., 2008), *L. fermentum* (GenBank NZ_AVAB01000110), *T. halophilus* (Satomi et al., 2008), and *T. muriaticus* (GenBank NC_918355). The sizes of these plasmids vary between 100 kb (*O. oenii*) and 23 kb (*T. muriaticus*). In the draft genome sequence of *L. saerimneri* 30a, one of the first lactobacillus species described to produce histamine (Rosenthaler et al., 1965), the HDC gene cluster is present in a 92-kb contig (GenBank ANAG01000006). As we did not find the *hdc* genes in the draft genome of *L. saerimneri* DSM 16094, we assume that the HDC locus is also encoded on plasmids in this species.

The alignment of these HDC gene cluster (Figure 3C) revealed that the sequences are highly conserved in the species *L. parabuchneri* and the other species that can also be present in food (*T. halophilus, T. muriaticus, L. hilgardii, L. sakei and O. oeni*). Interestingly, we found that the nucleotide sequence of the *hdcB* gene, which is predicted to catalyze the maturation of pyruvoyl-dependent histidine decarboxylase HDC (Trip et al., 2011) of *L. parabuchneri*, is highly different in *L. saerimneri, L. vaginalis, L. fermentum*, and *L. reuteri*. Especially, the first two-thirds of the *hdcB* gene show considerable less sequence identity than the last third.

Finally, we compared the conservation of the HDC gene cluster from different species to the general sequence conservation between *L. parabuchneri* and *L. buchneri*. We calculated the mean similarity of the nucleotide sequence between the four genes of the HDC gene cluster of *L. parabuchneri* and the HDC gene cluster from *T. halophilus, T. muriaticus*, and *L. hilgardii*. We found that the average DNA sequence identity is 82.5% of the genes of the HDC gene cluster. Compared to this the average sequence identity of the core genome of *L. parabuchneri* FAM21731 and *L. buchneri* NRRL B-30929 is 77.4%. We assume that the events of the separation of the species *L. parabuchneri* and *L. buchneri* and the gain of the HDC gene cluster lie chronologically rather close to each other.

## Discussion

We found strong evidence that the HDC gene cluster was introduced to the genome of *L. parabuchneri* by horizontal gene transfer and that the HDC gene cluster is located on a genomic island (HDC island, Figure 2A). This island—which has a different GC content than the other regions in the genome - is only present in strains that can synthesize histamine from histidine. Furthermore, we found that the HDC gene cluster is also conserved in species that are used in food production (Coton et al., 1998; Lucas et al., 2005; Satomi et al., 2011) and species that are adapted to the human body as habitat. Remarkably, the genes of the HDC gene cluster are more conserved between *L. parabuchneri, L. hilgardii, T. halophilus*, and *T. muriaticus* than the core genome between *L. parabuchneri* and *L. buchneri*. Also, the HDC gene cluster is located on plasmids in most of the strains of other species (Figure 2B), which facilitates horizontal gene transfer. However, the genes around this cluster are not syntenic to other known histaminogenic lactobacilli. Therefore, the donor species remains enigmatic. We propose that *L. parabuchneri* received the HDC gene cluster by horizontal gene transfer via a plasmid that eventually integrated into the chromosome. The finding that the HDC island has an increased GC-content (Figure 2) and is flanked by transposases (Figure 3A) supports this hypothesis. It was described in an earlier study that the plasmids with the HDC genes are unstable in *L. hilgardii* (Lucas et al., 2005) and in *O. oeni* (Lucas et al., 2008) and that the integration of the HDC gene cluster into the chromosome has a stabilizing effect on the genetic loci. However, we found six strains that miss the HDC island (Figure 2B) and assume that it was lost in a single event, as all six strains have similar neighboring regions to the HDC island (Figure 2B) but are different to *L. buchneri*.

We found that the strains of branch 2 originated from branch 3 but received a 400-kbp genomic region from a strain of branch 1 (Figure 4B). We could detect the pattern using Illumina reads and could confirm it using Ion Torrent technologies (Figure 4A). This finding is evidence that the 400-kbp region was inserted into the genome of a strain from branch 3 and originated from a strain from branch 1. The fact that the 400-kbp region in the strains of branch 1 and branch 2 are almost identical indicates that the integration occurred very recently, likely at the beginning of dairy farming. Interestingly, in rhizobia the horizontal transfer of a 500-kbp large element was described (Sullivan and Ronson, 1998) showing that even genomic elements of such sizes can be horizontally transferred.

It was shown that the HDC gene cluster can be used for pH regulation and energy production, i.e., that the decarboxylase (Rosenthaler et al., 1965) and carrier-mediated exchange generate a proton motive force (Molenaar et al., 1993). This is beneficial during the acidification and the ripening in the cheese making process. During the acidification, the habitat pH is lowered and bacteria that cannot adapt to this low pH will be neutralized or outgrown. During ripening free amino acids like histidine are more abundant than sugars and are therefore important energy sources.

The *hisS* gene of the HDC gene cluster that encodes a histidyl-tRNA synthetase gene is preceded by a histidine specific T-box. Martín et al. (2005) described a structural model of this T-box proposing a His (CAC) specifier sequence and demonstrated that the expression of *hisS* depends on the histidine concentration present in the medium. We found a second T-box upstream of locus FAM21731_00904 that also revealed a His (CAC) specifier sequence. Therefore, we assume that the expression of the downstream located operon is also modulated by the intracellular histidine concentration. This implies that the peptidase (FAM21731_00903) and the polypeptide transporter (FAM21731_00904) encoded in the operon are involved in the transport and degradation of histidine-containing peptides.

With regard to *hisS*, a second gene, represented by locus FAM21731_02461 and named *hisS2*, is present in the chromosome of *L. parabuchneri* FAM21731. The nucleotide sequence of *hisS2* showed 57% identity to *hisS* of the HDC island. The *hisS2* gene is preceded by a T-box and downstream lies the *aspS* gene (FAM21731_02460) that encodes an aspartyl-tRNA synthetase. T-box modulated *hisS-aspS* operons have identified in various bacteria including bacilli and lactobacilli (Gutierrez-Preciado et al., 2009). A characteristic of the T-box is the sequence GACAC in the specifier loop that is also present in the T-box of *L. parabuchneri* (Figure S3). Gutierrez-Preciado et al. (2009) suggested that this sequence mainly interacts with aspartyl-tRNA. Consequently, expression of the *hisS-aspS* operon would not be upregulated in the presence of uncharged histidyl-tRNA. This means when the intracellular histidine concentration drops e.g., due to the conversion to histamine, it could slowdown protein biosynthesis because not enough charged histidyl-tRNAs are present. Therefore, we assume that the additional *hisS* gene present in the HDC gene cluster is important to sequester sufficient histidine to sustain protein biosynthesis.

We also found other genes that could be involved in the amino acid metabolism or pH regulation on the HDC island. Among the seven genes related to ribose metabolism (Figure 3A) we identified two ribokinases. The ribokinases are required to convert ribose to ribose 5-phosphate (Neidhardt and Curtiss, 1996). These can be used for histidine synthesis (KEGG:M00026) (Ogata et al., 1999). Besides the genes involved in histidine mechanisms, we also found genes that are involved in the gluconate metabolisms on the HDC island. In particular, we identified four gluconate permeases on the island (Table 2) and other genes that are involved in the gluconate/ galactonate metabolism. We assume that these genes might have an effect on the pH, since the addition of zinc gluconate into the medium of lactic acid bacteria leads to enhanced acidification (Aquilanti et al., 2012). However, the connection of pH regulation and gluconate permeases is not completely clear and needs further investigations. Finally, our hypothesis is that many genes of the HDC island contribute to the adaptation of *L. parabuchneri* to the cheese and milk habitat.

We suppose that the emergence of the species *L. parabuchneri*, the introduction of dairy farming and the HDC island are connected. We assume that some strains of the common ancestor of *L. buchneri* and *L. parabuchneri* received the HDC gene cluster from another species during the adaptation process to the milk habitat. The HDC gene cluster provided these strains a growth advantage in the cheese habitat and led to a separation of the two species. Conversely, the strains without the HDC gene cluster remained in the original habitat and evolved to *L. buchneri*. After these events, there was a separation of *L. parabuchneri* into two braches; branch 1 with the HDC gene cluster and branch 3 that lost the HDC gene cluster. Finally, a strain from branch 3 received the large 400-kbp element from a strain from branch 1 and integrated it into the genome. Since important genes are specific for some strains and are shared within the *L. parabuchneri* species, our findings are in agreement with the distributed genome hypothesis (Ehrlich et al., 2005, 2010).

## Author contributions

DW, SI, HB, DWe, EE, and RB: Conceived and designed the study; SI and HB: Performed experiments; DW and RB: Performed bioinformatics analyses; DW, SI, and RB wrote the manuscript.

## Funding

This work was supported by the Canton of Bern to RB.

### Conflict of interest statement

The authors declare that the research was conducted in the absence of any commercial or financial relationships that could be construed as a potential conflict of interest.

## Supplementary material

Figure S1 **Synteny ortholog detection (SynOrF)**. The red and black dots represent genes located on two different bacterial genomes. The double lines indicate homology between genes. The arrows are edges in a graph. **(A)** The query gene of Genome_1 has two homologs in Genome_2. **(B)** The detailed construction of a graph is shown. The edges of the graph are the connections for each gene and its genomic neighbors up to the third degree. To evaluate the synteny, a score is calculated based on the number of neighbors of a homolog that are also homolog to the neighbors of the query gene **(C,D)**. Homologs with the highest score (≥2) are selected as synteny orthologs.

Figure S2 **Example of the detection of histidine and histamine**. The culture supernatants of *L. parabuchneri* strains were separated using thin-layer chromatography. The imidazol ring of histidine and histamine was visualized by immersing the plate into Pauly's reagent. The description of the lanes represents the strain name.

Figure S3 **T-box alignment**. The T-box (FAM21731_02461) upstream of the histidyl-tRNA synthetase (FAM21731_02461) and the aspartyl-tRNA synthetase (FAM21731_02460) was aligned with the histamine specific T-boxes of *Lactobacillus fermentum* [LAF_0755(hisZ)], *Lactobacillus plantarum* [lp_2561(hisZ)], and *Lactobacillus casei* [LSEI_1435(hisZ)] using the muscle web interface (http://www.ebi.ac.uk/Tools/msa/muscle/). The specifier codon of the histamine specific T-boxes is labeled with a green background and the GACAC pattern of FAM21731_02461 is labeled with a red background.

Table S1 **Genomes used for the phylogenetic tree**.
