# Supplementary material for: The Histidine Decarboxylase Gene Cluster of Lactobacillus parabuchneri Was Gained by Horizontal Gene Transfer and Is Mobile within the Species
Source: Front Microbiol. 2017 Feb 17;8:218. doi: 10.3389/fmicb.2017.00218 (PMC5313534; doi:10.3389/fmicb.2017.00218)
Supplement: Figure S1 — Synteny ortholog detection (SynOrF). The red and black dots represent genes located on two different bacterial genomes. The double lines indicate homology between genes. The arrows are edges in a graph. (A) The query gene of Genome_1 has two homologs in Genome_2. (B) The detailed construction of a graph is shown. The edges of the graph are the connections for each gene and its genomic neighbors up to the third degree. To evaluate the synteny, a score is calculated based on the number of neighbors of a homolog that are also homolog to the neighbors of the query gene (C,D). Homologs with the highest score (≥2) are selected as synteny orthologs. [file Image1.pdf]

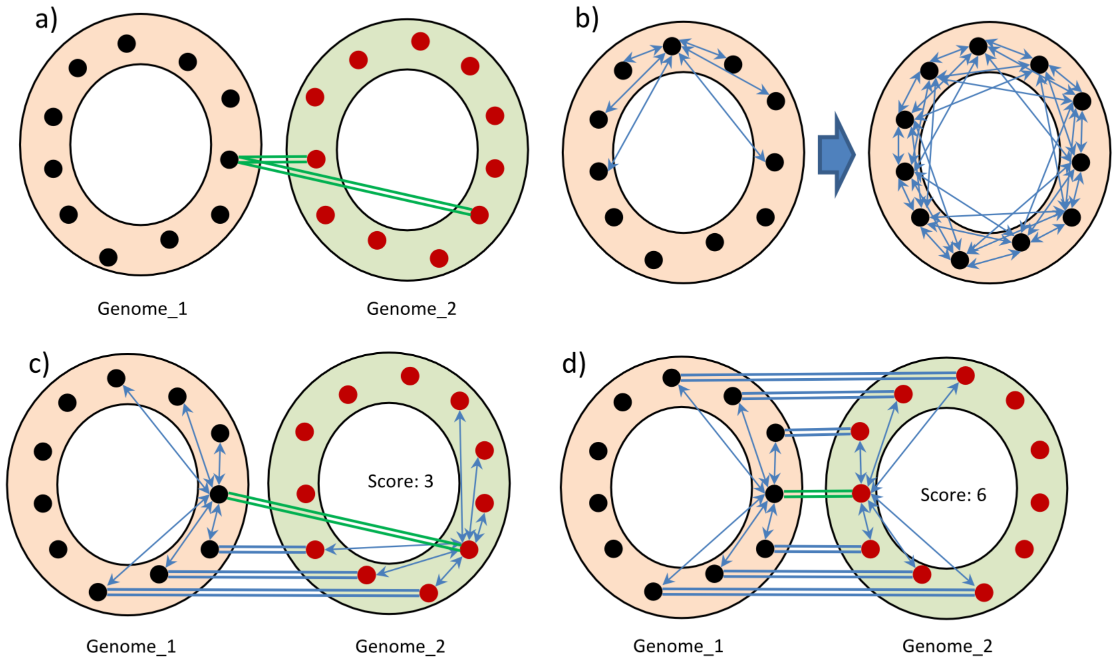

Figure S1: Synteny ortholog detection (SynOrF). The red and black dots represent genes located on two different bacterial genomes. The double lines indicate homology between genes. The arrows are edges in a graph. **a)** The query gene of Genome_1 has two homologs in Genome_2. **b)** The detailed construction of a graph is shown. The edges of the graph are the connections for each gene and its genomic neighbors up to the third degree. To evaluate the synteny, a score is calculated based on the number of neighbors of a homolog that are also homolog to the neighbors of the query gene **(c, d)**. Homologs with the highest score (>=2) are selected as synteny orthologs.
