# Supplementary material for: The Histidine Decarboxylase Gene Cluster of Lactobacillus parabuchneri Was Gained by Horizontal Gene Transfer and Is Mobile within the Species
Source: Front Microbiol. 2017 Feb 17;8:218. doi: 10.3389/fmicb.2017.00218 (PMC5313534; doi:10.3389/fmicb.2017.00218)
Supplement: Figure S2 — Example of the detection of histidine and histamine. The culture supernatants of L. parabuchneri strains were separated using thin-layer chromatography. The imidazol ring of histidine and histamine was visualized by immersing the plate into Pauly's reagent. The description of the lanes represents the strain name. [file Image2.pdf]

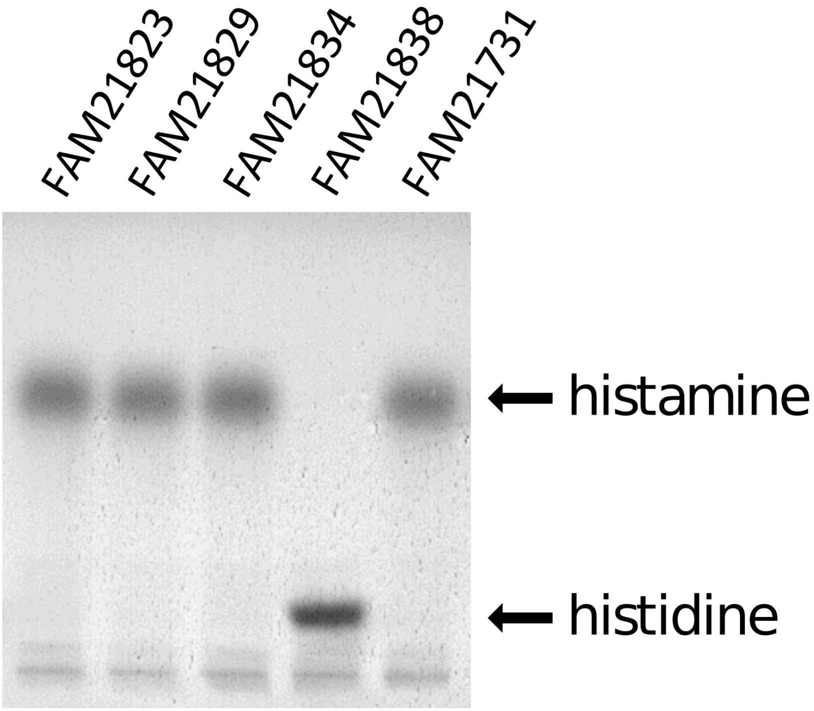

Figure S2: Example of the detection of histidine and histamine. The culture supernatants of *L. parabuchneri* strains were separated using thin-layer chromatography. The imidazol ring of histidine and histamine was visualized by immersing the plate into Pauly's reagent. The description of the lanes represents the strain name.
