# Supplementary material for: The Histidine Decarboxylase Gene Cluster of Lactobacillus parabuchneri Was Gained by Horizontal Gene Transfer and Is Mobile within the Species
Source: Front Microbiol. 2017 Feb 17;8:218. doi: 10.3389/fmicb.2017.00218 (PMC5313534; doi:10.3389/fmicb.2017.00218)
Supplement: Figure S3 — T-box alignment. The T-box (FAM21731_02461) upstream of the histidyl-tRNA synthetase (FAM21731_02461) and the aspartyl-tRNA synthetase (FAM21731_02460) was aligned with the histamine specific T-boxes of Lactobacillus fermentum [LAF_0755(hisZ)], Lactobacillus plantarum [lp_2561(hisZ)], and Lactobacillus casei [LSEI_1435(hisZ)] using the muscle web interface (http://www.ebi.ac.uk/Tools/msa/muscle/). The specifier codon of the histamine specific T-boxes is labeled with a green background and the GACAC pattern of FAM21731_02461 is labeled with a red background. [file Image3.pdf]

```
LAF_0755(hisZ)       --AATGAGTAAGGACCAGGAGAGTAGGAGTGGGGCTCC-CAAGCAGAGAGTCGCCG-GCG
FAM21731_02462       AC---------CGATAAAAAGAACAGTAGAAATTATCTTCAGCCAGAGAGCGGCCATTTG
LSEI_1435(hisZ)      ACATGAAGTTGTGATTAGAACAAGTAAGCTGATCCTGATCAA-CAGAGAGCGTCTA-TTG
lp_2561(hisZ)        ATAAACGGTTGTGATTAGAACAAGTAATTAAGTCTTCCTCATCCAGAGAGCCCGGG-TAG
                                 **  *  * *             *   **  *******         *

LAF_0755(hisZ)       CTGGGATGGCGATTGACCCCCGCTTCGAAC-CACACCTGGGAACTGGACGGCTGAACGG-
FAM21731_02462       ATGGAAGACCG--TGTTGATCATTTCGAAGTGACACTT----------------------
LSEI_1435(hisZ)      CTGAAAAGACG--CGATTAGCCAGCTGAACACACATCTATGAACTTGACGGCTGAACTA-
lp_2561(hisZ)        CTGAGAACCGGACAGACAGGCTTAACGAACACACATCTATGAACTGCTAAGGTGAACCAG
                      **  *    *   *     *     ***   ***  *

LAF_0755(hisZ)       ----AGTAGGTCGGCCCGGGACCA-CCCGTTAGCAGGGTTG-------------------
FAM21731_02462       -------------------------TTTGCTAATAATG----------------------
LSEI_1435(hisZ)      ----AGTAAGCTGTCACGCATCCGTTGCGTTATCACTGCTGCCAACTGCTGTTCGCAATT
lp_2561(hisZ)        TCTCATTAGCCTTAGCCGAATTGGTTTCGTTATCCAAGTTG-------------------
                                                 * **     *

LAF_0755(hisZ)       -----AAGTTAGCTTCAACGTGAGGCCCCTTTCGTGAGGGAG--------GGGTGAACGG
FAM21731_02462       -------GTTAATAGCGAAATGCTATTCG---TGCGAGCGTTATCCTTGAGAACTAATTA
LSEI_1435(hisZ)      GCTGACAGTTACTTGGCAGGTGAGGTTTAGGTCGCAAGAGCT-------AAACAAAATTG
lp_2561(hisZ)        ------ACTTGAT--CAACATGAGGTCTGCCGTGCGAACGGT--------AGATGAAATA
                             **       *  **           *  *  *               **

LAF_0755(hisZ)       AGGTGGAACCGTGCTCCTGTG-CCCTCTTGCA
FAM21731_02462       AGGTGGTACCGTGCGCATTG--CACCCTTGTC
LSEI_1435(hisZ)      AGGTGGTACCGCGGTCAAAAATCGCCCTCGGA
lp_2561(hisZ)        AGGTGGAACCACGCGTAAA---CGTCCTTTTA
                     ****** ***  *         *   **
```

Figure S3: T-box alignment. The T-box (FAM21731_02461) upstream of the histidyl-tRNA synthetase (FAM21731_02461) and the aspartyl-tRNA synthetase (FAM21731_02460) was aligned with the histamine specific T-boxes of *Lactobacillus fermentum* (LAF_0755(hisZ)), *Lactobacillus plantarum* (lp_2561(hisZ)) and *Lactobacillus casei* (LSEI_1435(hisZ)) using the muscle web interface (http://www.ebi.ac.uk/Tools/msa/muscle/). The specifier codon of the histamine specific T-boxes is labeled with a green background and the GACAC pattern of FAM21731_02461 is labeled with a red background.
